# The impact of a fast track area on quality and effectiveness outcomes: A Middle Eastern emergency department perspective

**DOI:** 10.1186/1471-227X-9-11

**Published:** 2009-06-17

**Authors:** Subashnie Devkaran, Howard Parsons, Murray Van Dyke, Jonathan Drennan, Jaishen Rajah

**Affiliations:** 1 Royal College of Surgeons in Ireland, Dubai Healthcare City, Dubai, United Arab Emirates; 2 Institute of Pediatrics, Sheikh Khalifa Medical City, Abu Dhabi, 51900, United Arab Emirates; 3 Institute of Emergency Medicine, Sheikh Khalifa Medical City, Abu Dhabi, 51900, United Arab Emirates; 4 Midwifery and Health Systems, University College Dublin, Belfield, Dublin 4, Ireland; 5 Institute of Pediatrics, Sheikh Khalifa Medical City, Abu Dhabi, 51900, United Arab Emirates

## Abstract

**Background:**

Emergency department (ED) overcrowding is a ubiquitous problem with serious public health implications. The fast track area is a novel method which aims to reduce waiting time, patient dissatisfaction and morbidity. |The study objective was to determine the impact of a fast track area (FTA) on both effectiveness measures (i.e. waiting times [WT] and length of stay [LOS]) and quality measures (i.e. LWBS rates and mortality rates) in non-urgent patients. The secondary objective was to assess if a FTA negatively impacted on urgent patients entering the ED.

**Methods:**

The study took place in a 500 bed, urban, tertiary care hospital in Abu Dhabi, United Arab Emirates. This was a quasi-experimental, which examined the impact of a FTA on a pre-intervention control group (January 2005) (n = 4,779) versus a post-intervention study group (January 2006) (n = 5,706).

**Results:**

Mean WTs of Canadian Triage Acuity Scale (CTAS) 4 patients decreased by 22 min (95% CI 21 min to 24 min, *P *< 0.001). Similarly, mean WTs of CTAS 5 patients decreased by 28 min (95% CI 19 min to 37 min, *P *< 0.001) post FTA. The mean WTs of urgent patients (CTAS 2/3) were also significantly reduced after the FTA was opened (*P *< 0.001). The LWBS rate was reduced from 4.7% to 0.7% (95% CI 3.37 to 4.64; *P *< 0.001). Opening a FTA had no significant impact on mortality rates (*P *= 0.88).

**Conclusion:**

The FTA improved ED effectiveness (WTs and LOS) and quality measures (LWBS rates) whereas mortality rate remained unchanged.

## Background

Emergency department (ED) overcrowding is becoming a ubiquitous manifestation representing an imbalance between the supply of medical resources and the demand by patients for quick and efficient service. It is a systemic and serious public health issue that affects industrialized countries all over the world [1-7]. Even though ED overcrowding has a multi-factorial origin that encompasses both internal and external factors, the use of EDs by non-urgent cases is also a contributing factor [1]. Therefore reducing the length of stay (LOS) and waiting times (WT) of non-urgent patients should contribute to a reduction in overcrowding.

A proportion of patient morbidity and mortality can be attributed to delays in early diagnosis and treatment, especially with time-sensitive diagnoses such as myocardial infarction, pneumonia, sepsis, and stroke [8]. Thus even mild conditions have the potential to become more serious if patients do not receive early medical care or they leave without being seen (LWBS) [9]. Finally, overcrowding is a cause of dissatisfaction among patients who wait the longest as well as a source of frustration among medical staff [1,10-14].

Since more than half the patients presenting to the ED having non-urgent conditions, an innovation like a fast track area (FTA) has the potential to reduce overcrowding [3]. A FTA is a recent innovation designed to reduce WTs of patients with minor injuries and illnesses [15]. The key principle of this system is that non-urgent patients are treated in a dedicated area by dedicated staff that has the competence to make discharge decisions, thereby preventing excessively long waits for such patients.

None of the previous studies reviewed were applicable to our institution. Firstly, our fast track is open 24 hours daily while all other studies had a limited operational time [7,16-21]. Secondly, none of the studies were set in a tertiary level, urban Middle Eastern hospital. Thirdly, with a few exceptions, most of the studies had very small and biased samples [7,21]. Finally, only one study, rigorously evaluated the effect of a fast track system on urgent patients [17].

The aim of this study was to determine if a FTA improved both effectiveness in service delivery (WTs and LOS) and quality measures (LWBS rates and mortality rates) for patients with minor injuries and illnesses classified according to the Canadian Triage Acuity Scale 4 and 5 (CTAS 4/5), without delaying the care of urgent patients (CTAS 2/3).

## Methods

### Study Setting and Design

This study took place in a 500 bed urban tertiary care general hospital, Sheikh Khalifa Medical City, in the United Arab Emirates (UAE). The public emergency care facility serves residents of Abu Dhabi (capital city of the UAE) and surrounding areas. In 2005, the ED had an annual census of approximately 70 000 patients. The study population consisted of adult and pediatric patients (defined as patients less than 12 years old as per hospital policy). The ED included a three-bed resuscitation area, and 15 monitored acute treatment beds (total of 18 ED beds) in the pre-fast track period and 7 additional FTA beds after the intervention (total of 25 beds). This was a single center study of ED department services at our hospital which provides all major medical, surgical and pediatric disciplines.

The FTA was opened in February 2005. All patients entering the ED were seen by triage nurses and classified according to the Canadian Triage and Acuity Scale (CTAS) [22]. The low acuity patients (CTAS 4 and 5) were then treated, referred or discharged by the physician from the FTA. Urgent patients (CTAS 2 and 3) were seen in the main ED. The CTAS is a 5 level triage scale based primarily on the patients presenting complaint and physiologic parameter. The CTAS guidelines are to ensure timely access to physician assessment on the basis of triage acuity level. A patient in CTAS 1 (resuscitation) requires immediate attention. CTAS 2 (emergent) should be seen within 15 minutes. CTAS 3 (urgent) should be seen within 30 minutes and the non urgent, CTAS 4 and 5 should be seen within 60 minutes and 120 minutes respectively. The typical patient in CTAS 4 and 5 is ambulatory, does not need extensive investigation and contributes to < 10% of total admissions.

The characteristics of our FTA are as follows: It has seven beds, is operational 24 hours a day, is staffed by either one or two Arabic speaking doctors at any time (of which 40% are house-officers and 60% are specialists with ED experience but no formal certification) depending on peak visits, sees only CTAS 4/5 (non-urgent) patients and performs only point of care laboratory testing e.g. pregnancy tests, urine dipsticks, glucose and chest X rays. The case mix of our patients can be inferred by examining the percentage of patients in the different CTAS categories. The construction of the FTA was as an additional resource and was built adjacent to the old ED. However the staffing from a nursing and physician perspective was by realignment of the current resources, without new staff being recruited. At all times there were 2 full time nursing equivalents to staff the 7 FTA beds. The main ED is typically consultant driven with Western trained staff. Junior staff who worked in the main ED in 2005, were assigned to the FTA in 2006. Being Arabic speaking circumvented the use of a translator in this area.

**Figure 1:**
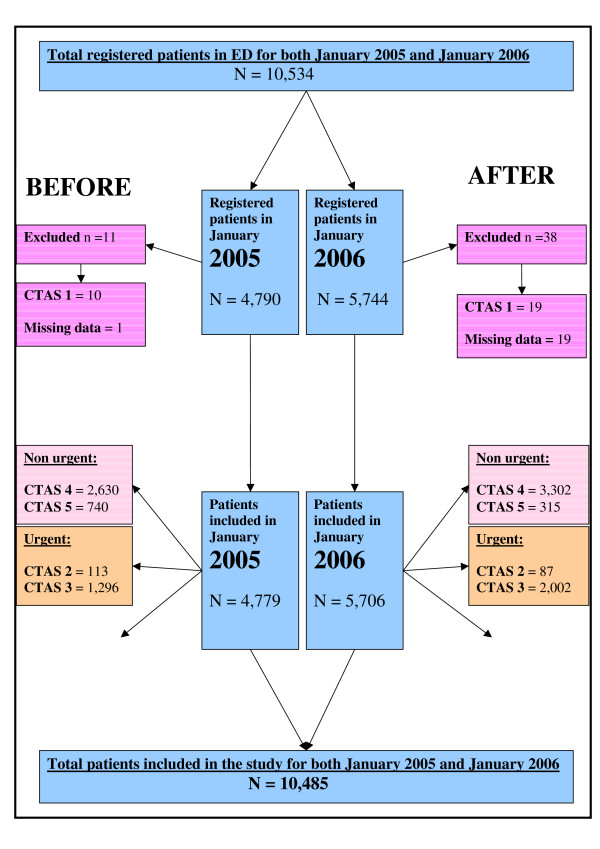
**A schematic summary of the number and disposition of study participants**.

**Figure 2:**
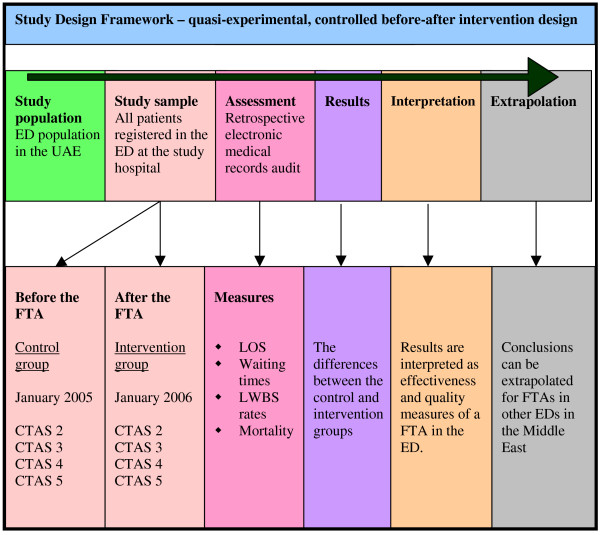
**Framework of this study's design**.

This study used a non-randomized, quasi-experimental, before-after intervention design with a historical control group to assess the performance of a FTA in an ED. Figure 1 depicts the disposition, sample sizes and triage categories of the patients, whereas Figure 2 depicts the framework of this study's design. A retrospective data analysis was performed of all patients registered at the ED before (January 2005) and after (January 2006) the opening of a new FTA.

### Operational Definitions of Terms

For the purposes of this study the following definitions were used:

◆ Waiting time (Time to physician assessment) – defined as the time interval from registration to initial contact by a physician [17]. This is expressed in minutes.

◆ Length of Stay (LOS)- defined as the time interval from registration to discharge disposition time [3,23,24]. This is expressed in minutes.

➢ For admitted patients: Arrival time to admission orders.

➢ For discharged patients: Arrival time to physical discharge.

➢ For transferred patients: Arrival time to transfer orders.

◆ Discharge Time – The time of physical departure of a discharged patient from the ED treatment area.

◆ Left without being seen (LWBS) rate – the number of patients who have undergone a triage assessment and code allocation but subsequently chose to leave before medical assessment [6]. This is expressed as a percentage of monthly ED visits.

◆ Monthly mortality rate – the number of patients each month who are pronounced dead in the ED [18]. This is expressed as a percentage of monthly ED visits.

*The following criteria were used for patient sampling*:

#### Inclusion criteria

1. All patients (pediatrics and adults) presenting to the ED in January 2005 (pre-FTA) and January 2006 (post-FTA), which included:

• CTAS 4 and 5 (non-urgent) patients for primary objective of the study.

• CTAS 2 and 3 (urgent) patients for the secondary objective of the study.

#### Exclusion criteria

1. CTAS 1 (emergent) patients as they are seen immediately.

2. Patients with missing data.

Interval sampling of the population from identical months (January) was chosen to eliminate the confounding variable of seasonal variation. This month excluded the month of Ramadan (the Islamic month of fasting), school holidays and the summer vacation months and therefore precluded periods where variation in ED attendance was expected. These factors were potentially important confounding variables. The one year time frame between study periods also allowed for stabilization of the new FTA and acted as a "wash-out" period.

### Data collection methods

Data was retrospectively extracted by the researcher and data analyst from the routine hospital information system for each patient. The data analyst who had earlier captured the original data was blinded to the hypothesis since this was a retrospective study. The computerized system was built on a Microsoft sequel server with the capability to access ordered interventions and results. A standardized data collection spreadsheet was used. There was no change in the health information system during both study periods. The key times were hand written and entered at the time of discharge onto a Microsoft Excel spreadsheet.

Data was collected retrospectively from the electronic hospital system for all patients registered at the ED before and after the opening of the FTA (i.e. January 2005 and January 2006 respectively).

Data validation consisted of checking for incomplete or missing data and correlating data items. Range checks were done to identify outliers in the data. The accuracy of all fields in the data was cross checked to ensure that all transfers, recodes and calculations were correct. Double checking against paper charts was performed by the data analyst with invalid or excessive WTs and randomly with 1% of patient records.

The data entered for each study patient comprised of the following information: date of arrival to the ED, arrival time to the ED, WT, LOS, LWBS, discharge time, died or survived, the triage category and hospital disposition.

### Statistics

Data analyses were performed using MedCalc for Windows, version 9.20 (MedCalc Software, Mariakerke, Belgium). Data screening and a check for the plausibility and distribution of data were conducted before performing descriptive statistics to ensure that the data met the statistical assumptions necessary for data analysis.

The outcome measures of the study were divided into effectiveness measures (WTs and LOS) and quality measures (LWBS and mortality rate). Univariate descriptive analysis was computed for the effectiveness measures and expressed as the mean and standard deviation. Bivariate analyses were used to determine differences in the effectiveness measures of WTs and LOS between the control and intervention groups. The independent sample *t*-test was used to calculate the differences in the mean WTs and mean LOS between the two study groups and the differences were expressed as 95% confidence intervals. With a large sample size (as in our study), the independent sample *t*-test is robust and the *P *value will be nearly correct even if a population is far from Gaussian [25].

Quality measures (mortality and LWBS rates) were analyzed using frequencies and proportions. The differences in the proportions were calculated using Chi-square tests and expressed as 95% confidence intervals. All hypotheses testing were two-tailed. A *P *value of < 0.05 was considered statistically significant.

The sample size was determined on the basis of an *a priori *power calculation. Using previously published data from and pilot data from our ED to estimate standard deviations, power calculations were made at alpha = 0.05 (type 1 error) and beta = 0.10 (type 2 error) [18]. The sample size needed to detect a change in the waiting time of 5 minutes, 10 minutes and 15 minutes was 204, 362 and 814 patients respectively. The sample size of our study was approximately 10,485 (4,779 patients before the FTA and 5,706 after). Our study was therefore adequately powered.

### Ethics

Prior to data collection, Institutional Review Board ethics approval was obtained from the study hospital. Ethical principles were applied to the storage, security, destruction, and retention of data. Data collection, analysis and storage were in accordance with the Data Protection Act of 1988 [26].

## Results

The study population consisted of mainly UAE nationals as this was the mandate of our hospital during the time of the study. Table 1 shows the baseline characteristics of the study sample. Pediatric patients accounted for a substantial proportion (about 40%) of the ED visits, during both study periods. The percentage of missing data for 2005 was 0.000021% (n = 1) while the missing data for 2006 was 0.0033% (n = 19).

**Table 1:** Baseline characteristics of study participants before and after FTA implementation

**Variable**	**Before FTA (Jan 2005)** **n-4, 790** **n (%)**	**After FTA (Jan 2006)** **N = 5, 744** **N (%)**
Male (%)	2,730 (57%)	3,504 (61%)
Females (%)	2,060 (43%)	2,240 (39%)
Adult (%)	2,826 (59%)	3,561 (62%)
Pediatric (%)	1,964 (41%)	2,183 (38%)
		
Non urgent		
CTAS 5	740 (15.5%)	315 (5.5%)
CTAS 4	2,630 (54.9%)	3,302 (57.7%)
		
Urgent		
CTAS 3	1,296 (27.1%)	2,002 (35.0%)
CTAS 2	113 (2.4%)	87 (1.5%)

Statistically significant reductions in both mean WTs and mean LOS of non-urgent (CTAS 4/5) patients were found after the implementation of a FTA (Tables 2 and 3). A statistically significant reduction in the LWBS rates was seen post-FTA implementation, whereas mortality rates were unchanged (Table 4). In addition the FTAs' impact on urgent patients was favorable as the results showed a statistically significantly decrease in the mean WTs of urgent patients (CTAS 2/3) and a statistically significant decrease in the mean LOS of CTAS 2 patients (Tables 2 and 3).

**Table 2:** Mean waiting times (minutes) for CTAS 2, 3, 4 and 5 compared before and after the opening of the fast track)

			**Independent samples** ***t*-test**		
			
**Outcome measure** **Waiting times**	**Before FTA** **2005** **Mean (SD)**	**After** **FTA** **2006** **Mean** **(SD)**	**Test** **Statistic** ***t*value**	**Difference** **(95% CI of difference)**	***P *value** **Two** **tailed**
CTAS 2* WT (min)	13.83	7.81	-2.09	-6.1(-11.7 to -0.3)	= 0.038
	(22.42)	(16.79)			
CTAS 3* WT (min)	29.04	24.75	-4.20	-4.2(-6.3 to -2.3)	<0.001
	(29.45)	(30.30)			
CTAS 4** WT (min)	45.79	23.23	-24.25	-22.6(-24.4 to-20.7)	<0.001
	(45.59)	(23.78)			
CTAS 5** WT (min)	48.20	19.80	-6.31	-28.4 (-37.2 to -19.6)	<0.001
	(76.15)	(27.75)			

WT: waiting time of the patient, "Urgent patients, **Non-urgent patients,

P < 0.05 considered statistically significant

**Table 3:** Mean LOS (minutes) for CTAS 2, 3, 4 and 5 compared before and after the opening of the fast track

			**Independent samples** ***t*-test**		
			
**Outcome measure** **LOS**	**Before FTA** **FTA** **2005** **Mean** **(SD)**	**After** **FTA** **2006** **Mean** **(SD)**	**Test** **Statistic** ***t*value**	**Difference** **(95% CI of difference)**	***P *value** **Two** **tailed**
CTAS 2* LOS (min)	188.71	149.51	-2.43	-39 (-71 to -7)	= 0.016
	(124.18)	(97.21)			
CTAS 3* LOS (min)	155.52	154.42	-0.30	-1(-8 to 6)	= 0.77
	(110.57)	(100.68)			
CTAS** LOS (min)	104.65	76.84	-13.86	-28(-32 to -24)	<0.001
	(82.14)	(72.05)			
CTAS 5** LOS(min)	75.11	43.48	-8.19	-32(-39 to -24)	<0.001
	(62.36)	(42.71)			

LOS: length of stay, *Urgent patients, **Non-urgent patients,

P < 0.05 statistically significant

**Table 4:** Quality measures of LWBS rates and mortality rates compared before and after the fast track area opened

			**Chi-square test**		
			
**Outcome measure**	**Before FTA** **FTA** **(2005)**	**After FTA** **(2006)**	**Chi-square value**	**Difference** **(95% CI of difference)**	***P *value**
Number of patients LWBS (%)	226 (4.72%)	41 (0.71%)	168.47	4% (3.37 to 4.65)	P < 0.001
					
Number of patients Deceased (%)	19 (0.397%)	25 (0.44%)	0.022	0.038% (-0.23 to 0.29)	P = 0.88

LWBS: Left without being seen by the doctor

P < 0.05 considered statistically significant

The percent of patients in CTAS 4 and 5 admitted from the ED into the inpatient department was 2%. The case mix included patients without circulatory and respiratory difficulties, who were ambulatory, who were unlikely to require intravenous fluids or medications and whose expected treatment time was 1 hour or less. It also excluded children < 1 year. The vast majority of patients (>60%) seen in both 2005 and 2006 were in the non urgent (CTAS 4/5) category.

By breaking the 24 hour day into 4 time cycles i.e. 00:00–06:00; 06:00–12:00; 12:00–18:00; 18:00–24:00 we found that the FTA impact persisted during every time cycle. This was notwithstanding the fact that the busiest flow of patients was between 18:00-06:00 where patient numbers were approximately double the earlier period.

## Discussion

Both WTs and LOS in CTAS 4 and 5 decreased by approximately 30 minutes after the opening of the FTA. This represented a 50% improvement in the WTs and a 30% – 40% improvement in the LOS. These decreases are both statistically significant and clinically important. In the context of time sensitive diagnosis and treatment, a few minutes may represent a crucial difference between life and death or significant morbidity. This improved flow through the ED was accomplished notwithstanding the 19.9% increase in the overall ED census in general and a 7% increase in CTAS 4/5 in particular (Table 2 and Table 3) in January 2006. This impact on non-urgent patients was noteworthy as two thirds of the sample population was in the non-urgent triage category (Figure. 1).

One year after the FTA was implemented, the quality of care had improved as measured by a commonly used indicator i.e. LWBS rate. The LWBS rate was reduced from 4.71% to 0.71% resulting in a relative reduction of 85%. This suggests that a FTA with improvements in WTs and LOS can have a large impact on the vulnerable LWBS population. Mortality was unchanged implying that the care of the emergent and urgent patients did not suffer as a result of the opening of the fast track.

There were some notable baseline differences between both study periods. There was a slight male predominance in the sample which is likely due to random variation. The 4% drop in the proportion of females in the post intervention group cannot be explained but may also be a manifestation of random variation. There was a 7.9% increase in the percentage of patients in the CTAS 3 group after the FTA was implemented. A possible explanation for this our hospital accepting more trauma cases resulting in an increase in the percentage of urgent (CTAS 3) patients presenting to the ED in 2006. Finally, the percentage of the CTAS 5 patients varied between both study periods (15.5% vs. 5.5%). This may represent an element of triage misclassification in the grey zone between CTAS 4 and 5. The absolute number of non urgent patients (combined CTAS 4 and 5) seen varied very little between both study periods (Table 1).

Although this study has confirmed the findings of previous studies, most of them relate to EDs in the United States of America, the United Kingdom and Australia [7,16-21]. A clinically significant element of this study's results was that the mean LOS and mean WTs decreased along with a clinically important decrease in the corresponding standard deviations (refer to Table 2 and Table 3). This finding is in contrast to other studies where the standard deviations and confidence intervals were wider [16,17,19]. Anecdotally, the narrow variation has impacted positively, leading to a reduction in the number of patient complaints. Unlike most other studies which required additional staffing resources, we achieved our goal by realignment of staff. Other unique features of the fast track area is that it was culturally sensitive (Arabic speaking doctors) and operated on a continuous 24 hour cycle. We did not examine a rapid entry and accelerated care at triage unlike a recent large trial which altered processes and revised their health informatics technology [27].

This study has also demonstrated that the opening of a FTA had no detrimental impact on the WTs and LOS of patients with serious injuries and illnesses. Both the mean WTs of CTAS 2 and CTAS 3 patients decreased (Table 2). LOS also decreased in the post-intervention CTAS 2 group. These improvements were unexpected because the FTA is designed to expedite the care of non-urgent patients only. This improvement may have occurred for a number of reasons. Firstly, since the FTA reduced overcrowding in the ED waiting room by diverting non-urgent patients to a separate treatment area, it may have given staff more physical space as well as a less distracting environment to focus their activities. Secondly, the frenetic environment of the overcrowded ED has a negative effect on physician productivity. At a certain limit of patients, productivity declines and patient care is compromised [1]. Presumably, a decrease in overcrowding may have improved physician productivity.

One method to mitigate the impact of low acuity patients on ED overcrowding is to triage them to care elsewhere (walk-in clinics, same-day or next-day visits with a primary care provider, etc). However, it is both medically unsafe and financially unnecessary to create barriers to ED care for low-acuity patients. It is more appropriate to identify the needs of this subset of patients and to subsequently tailor the delivery of resources to meet these needs. As noted by the Institute for Healthcare Improvement, strategies that reduce operational cycle times and improve patient flow are critical to accomplish this [28]. This is the basis for the development of a FTA for low-acuity patients that many hospitals have initiated.

## Limitations

Randomization of ED patients with acute medical problems is difficult due to the ethical constraints and administrative constraints in such patients. Similar to our study looking at ED FTAs, the predominant research design of prior studies was quasi-experimental. We attempted to remove threats to the internal validity of our study, which is the main limitation of this design [29-31]. Firstly, the sample size of this study was large (n = 10,485) in relation to previous studies [16-18]. The large sample size mitigated against the outcomes being attributed to regression to the mean. Secondly, there were many outcomes that varied statistically with the intervention [29,32]. The four outcome variables were WTs, LOS, LWBS rates and mortality rates. Thirdly, our comparison groups are matched according to triage category to eliminate confounding variables related to illness severity. Fourthly, this study was designed with a one year "wash-out" period, allowing for stabilization of the FTA operation. Fifthly, the same months (i.e. January 2005 and January 2006) were compared to eliminate seasonal/cyclic variation. Finally, there was little change in other potential confounding variables like staffing ratios, bed-patient ratios and the availability of equipment [29]. Since this was a retrospective analysis, nurses and clerical staff who inputted the data were unaware that a study would be conducted, thus avoiding the Hawthorne effect (i.e. people perform differently by being aware of an ongoing investigation).

The studies generalizability is limited to similar ED's servicing a large proportion of pediatric patients (40%) and who see a high proportion of low acuity patients (65%–70%). Being a retrospective study, we did not measure other more sensitive measures of quality like timeliness of medications, return visits, quality variance reports and subsequent admissions. Also a time series analysis to detect monthly variability was impractical as we lacked appropriate historical data prior to the intervention of the FTA.

## Conclusion

This study adds a Middle Eastern perspective of the FTA's impact on non urgent patients, in a tertiary hospital with a mixed caseload which included pediatric and adult patients. A fast track system appears to be an effective method by which a busy ED can efficiently maintain patient flow in light of restricted resources, space constraints, limited manpower, and an escalating patient census.

## Competing interests

The authors declare that they have no competing interests.

## Authors' contributions

SD conceived on the study, participated in its design and coordination, acquisition of the data, drafting of the manuscript and analysis and interpretation of the data. HP participated in the study design and critically reviewed the script at all stages for important intellectual content. MVD helped with the acquisition of data, provided administrative support and reviewed the manuscript critically. JD was responsible for study supervision and drafting of the manuscript. JR helped with acquisition of the data, analysis and interpretation of the data, critical revision of the manuscript and provided statistical expertise. All authors read and approved the final manuscript.

## Pre-publication history

The pre-publication history for this paper can be accessed here:


